# Nationwide Implementation of Unguided Cognitive Behavioral Therapy for Adolescent Depression: Observational Study of SPARX

**DOI:** 10.2196/66047

**Published:** 2025-08-25

**Authors:** Terry Fleming, Mathijs Lucassen, Chris Frampton, Varsha Parag, Chris Bullen, Sally Merry, Matthew Shepherd, Karolina Stasiak

**Affiliations:** 1 Te Kura Tātai Hauora School of Health, Victoria University of Wellington, PO Box 600, Wellington, 6140, New Zealand, 64 7961680776, 64 800 04 04 04; 2 Department of Epidemiology and Biostatistics, The University of Auckland, Auckland, New Zealand; 3 School of Health and Psychological Sciences, City St George's, University of London, London, United Kingdom; 4 Psychological Medicine, University of Otago, Christchurch, New Zealand; 5 National Institute for Health Innovation (NIHI), The University of Auckland, Auckland, New Zealand; 6 School of Population Health, The University of Auckland, Auckland, New Zealand; 7 Department of Psychological of Medicine, The University of Auckland, Auckland, New Zealand; 8 School of Psychology, Massey University, Auckland, New Zealand

**Keywords:** therapy, digital health, serious games, self-help, implementation, cognitive behavioral therapy, CBT, internet-based cognitive behavioral therapy, iCBT, depression, teenager, youth, adolescent, SPARX, mHealth, mobile health

## Abstract

**Background:**

Internet-based cognitive behavioral therapy (iCBT) interventions are effective in clinical trials; however, iCBT implementation data are seldom reported.

**Objective:**

The objective of this study is to evaluate uptake, adherence, and changes in symptoms of depression for 12‐ to 19-year-olds using an unguided pure self-help iCBT intervention (SPARX; Smart, Positive, Active, Realistic, X-factor thoughts) during the first 7 years of it being publicly available without referral in Aotearoa New Zealand.

**Methods:**

SPARX is a 7-module, self-help intervention designed for adolescents with mild to moderate depression. It is freely accessible to anyone with a New Zealand Internet Protocol address, without the need for a referral, and is delivered in an unguided “serious game” format. The New Zealand implementation of SPARX includes 1 symptom measure—the Patient Health Questionnaire adapted for Adolescents (PHQ-A)—which is embedded at the start of modules 1, 4, and 7. We report on uptake, the number of modules completed, and changes in depressive symptoms as measured by the PHQ-A.

**Results:**

In total, 21,320 adolescents aged 12 to 19 years (approximately 2% of New Zealand 12‐ to 19-year-olds) registered to use SPARX. Of these, 63.6% (n=13,564; comprising n=8499, 62.7% female, n=4265, 31.4% male, and n=800, 5.9% another gender identity or gender not specified; n=8741, 64.4% New Zealand European, n=1941, 14.3% Māori, n=1202, 8.9% Asian, n=538, 4.0% Pacific, and n=1142, 8.4% another ethnic identity; mean age 14.9, SD 1.9 years) started SPARX. The mean PHQ-A at baseline was 13.6 (SD 7.7) with 16.1% (n=1980) reporting no or minimal symptoms, 37.4% (n=4609) reporting mild to moderate symptoms (ie, the target group) and 46.7% (n=5742) reporting moderately severe or severe symptoms. Among those who started, 51.1% (n=6927) completed module 1, 7.4% (n=997) completed at least 4 modules, and 3.1% (n=416) completed all 7 modules. The severity of symptoms reduced from baseline to modules 4 and 7. Mean PHQ-A scores for baseline, module 4, and module 7 for those who completed 2 or more assessments were 14.0 (SD 7.0), 11.8 (SD 7.9), and 10.5 (SD 8.5), respectively; mean difference for modules 1-4 was 2.2 (SD 5.7; *P*<.001) and for modules 1-7 was 3.6 (SD 7.0; *P*<.001). Corresponding effect sizes were 0.38 (modules 1-4) and 0.51 (modules 1-7).

**Conclusions:**

SPARX reached a meaningful proportion of the adolescent population. The effect size for those who engaged with it was comparable to trial results. However, completion was low. Key challenges included logistical barriers such as slow download speeds and compatibility with some devices. Ongoing attention to rapidly evolving technologies and engagement with them are required. Real-world implementation analyses offer important insights for understanding and improving the impact of evidence-based digital tools and should be routinely reported.

## Introduction

Adolescent depression is common and disabling [1,2]. Over 20% of Aotearoa New Zealand secondary school adolescents reported clinically significant depression symptoms in 2019 [3]. Evidence-based talking therapies, including cognitive behavioral therapy (CBT), are the recommended first line of treatment for mild to moderate depression [4]. However, 50%‐80% of young people with symptoms do not get treatment [5]. Internet-based cognitive behavioral therapy (iCBT) programs have been developed, partly in response to these challenges, and have been shown to be effective for the treatment of adult and adolescent depression [6].

Co-authors (TF, KS, ML, MS, and SM) developed a computerized CBT intervention for adolescent depression called “SPARX” (Smart, Positive, Active, Realistic, X-factor thoughts). SPARX performed at least as well as treatment as usual in a non-inferiority randomized controlled trial (RCT) with 187 adolescents seeking help for low mood or depression in primary care [7], and it was at least as effective for indigenous Māori adolescents as for non-Māori [8]. In smaller trials, SPARX was effective compared to waitlist control among adolescents excluded from mainstream education [9]; promising in an adapted “Rainbow version” open trial among sexual and gender minority youth [10]; and appealing to a range of youth [11-13], although a Dutch version of SPARX was not more effective than a monitoring control or group-based CBT in an RCT in classroom settings [14]. A revised version of SPARX (SPARX-R), adjusted to include those without depression, was not successfully implemented in a youth justice setting, where participants did not attend the youth justice program regularly and few tried SPARX-R [15]; however, it was effective for the prevention of depression at 6 months follow-up in a large RCT (n=540) conducted in Australian secondary schools [16].

In 2014, the New Zealand Ministry of Health funded SPARX as an unguided self-help iCBT intervention. It was targeted to adolescents with mild to moderate depression but was freely available to anyone with a New Zealand Internet Protocol (IP) address with no referral required. In this paper, we explore outcomes for 12‐ to 19-year-olds who registered for SPARX from 2014 to 2021; the first 7 years of SPARX being nationally available. There are some complexities. SPARX was originally trialed on CD-ROMs; rapid transition to web-based delivery was required for national rollout. For the first years of national delivery, SPARX was only available via desktop or laptop computers (not smartphones or tablets), so users could register but not begin on a mobile device, and users were required to download a large file, which was not possible for some (eg, due to firewalls or problematic internet speeds).

Despite, and in part *becaus*e of, operational challenges, it is valuable to examine implementation findings. While there are many trials of evidence-based iCBT programs in the peer-reviewed literature, there are few studies of how these programs perform in real-world settings [17,18]. In a 2018 systematic review, there were peer-reviewed findings from just 7 self-help interventions for depression, anxiety, or enhancement of mood as implemented in real-world settings [17], with these reporting much lower rates of retention than in trials of the same interventions. Moreover, routinely collected internet traffic data indicates median retention in publicly available mental health app-based programs is less than a quarter of that for the same programs evaluated in trials [18]. While there may be user benefits from even brief engagement [18], and, if initial uptake is high, even low rates of completion can have population impact, these data call for examination.

The research-to-implementation distinction is not unique to digital tools. For example, while 6 or more sessions of face-to-face CBT are recommended for initial treatment in many clinical guidelines [4], in practice, there are considerable barriers, including a lack of services, limited resources, and extensive waiting lists [19]. Internet-based interventions offer the ability to examine distinctions between ideal and real implementation, given the opportunities afforded by routine data collection in web-based delivery. In this paper, we report on the uptake, engagement, and impact of SPARX by 12‐ to 19-year-olds in its implementation in the “real world” as a pure self-help (ie, unguided) intervention.

## Methods

### Study Design

This was a purely observational retrospective cohort study of national implementation of SPARX iCBT in Aotearoa New Zealand. There were no recruitment or study procedures beyond analysis of anonymous self-reported data and automatically collected analytics for 12‐ to 19-year-old users who registered to use SPARX via the New Zealand website between April 1, 2014, and September 30, 2021. There were no data collected outside of the SPARX program.

### Ethical Considerations

All users, including minors, provided their own consent to use SPARX and for anonymous data to be analyzed for evaluating and improving the program. Synchronous human-delivered support via toll-free telephone, text, or messaging services was available free of charge. Participants did not receive compensation or payments.

Parent or guardian consent was not required for the use of SPARX as implemented in New Zealand. This decision was made by the funder (the New Zealand Ministry of Health) and provider (the New Zealand Institute of Health Innovation at the University of Auckland) in collaboration with national clinical and governance groups overseeing the SPARX rollout. The decision to allow minors to self-consent was grounded in clinical and ethical considerations. The risks associated with untreated depression and other mental health issues in young people are well documented [1] and parent engagement is a significant barrier to treatment for some [20,21]. Recent New Zealand data underscore this point. Among male secondary school students who had attempted suicide in the past 12 months, only 36% had talked to a parent or family member about feeling bad [22]. Upon registration for SPARX, users agreed to “get extra help” if SPARX was not helping or was not sufficient for their needs. The website and SPARX program included messaging encouraging young people to talk with family members and others, along with guidance on how to do so and where and how to access further support.

Parental or guardian consent was also not required for data to be used in this analysis. Requiring additional consent for the use of routinely collected data can introduce selection bias in observational studies [23] and the New Zealand National Ethical Standards for Health and Disability Research and Quality Improvement [24] and Health Information Governance Guidelines [25] allow for the use of nonidentifiable health information such as this for research and evaluation purposes.

The study was approved by the New Zealand Health and Disability Ethics Committee (reference: 15/NTB/183) and is reported using STROBE (Strengthening the Reporting of Observational Studies in Epidemiology) criteria [26].

### Participants and Setting

Automatically collected analytics from all 12‐ to 19-year-olds who registered for SPARX as implemented between April 1, 2014, and September 30, 2021, were analyzed. There were no exclusion criteria beyond age group, registration, and time period. Given the observational nature of this research, no predetermined sample size was calculated. Instead, the size of the study was based on the number of adolescent users accessing SPARX from the start of the New Zealand-wide rollout of the program (April 1, 2014), to just prior to the use of SPARX 2.0 (ie, a new iteration of the intervention, September 30, 2021).

Participants could register for SPARX on any device with internet access and could open SPARX on a PC or laptop, or, from 2018, on a mobile device from any location via a New Zealand IP address. While there were barriers such as internet firewalls and download speeds (described under “Implementation Model”), the SPARX website was available more than 99.9% of the time, and users could register and begin SPARX at any time during the 7-year study period. Exposure varied from registration only to completion of all 7 levels of SPARX with no limitations on time taken.

### Intervention

The SPARX program has been described elsewhere [7]. In brief, it is a structured iCBT program that includes interpersonal skills and emotion regulation content. SPARX uses a bi-centric frame of reference involving both explicit information giving and discovery or implicit learning [7]. A guide (virtual therapist) introduces the program and “speaks” directly with the user at the start and close of each of the 7 modules, with scripted dialogue to support rapport, hope, engagement, consolidation, and generalization of therapeutic content. The core part of each module takes place in a game-like setting, where the user takes on challenges and solves problems to “restore the balance” and solve problems in a fantasy world, allowing them to discover and rehearse CBT concepts and skills in a playful way as they progress. At the end of each module, users return to the guide to consolidate and review key concepts and to encourage the application of concepts and skills in everyday life with quizzes, open text questions, and reflective exercises.

The therapeutic content of SPARX has also been described previously [7]. In summary, module 1 focuses on building virtual rapport, with caring and welcoming words from the guide and the expression of hope and expectations of improvement (even if multiple steps are required). Psychoeducation about variation in mood and depression and how moods and feelings change is provided. Module 1 also includes how to find additional forms of support, and it introduces relaxation breathing. Module 2 pertains to behavioral activation and key prosocial communication skills. Module 3 focuses on dealing with strong emotions, while module 4 explores problem-solving. Modules 5 and 6 relate to recognizing and challenging unhelpful thoughts and growing realistic positive thoughts and action thinking. Finally, module 7 integrates the included skills and concepts and includes additional strategies and resources for managing persistent challenges.

### Implementation Model

In 2014, the SPARX research team, in collaboration with the National Institute for Health Innovation at the University of Auckland, was the successful applicant for New Zealand Ministry of Health funding to deliver a youth e-therapy. A pure self-help delivery model, via a publicly available website with phone or text or web chat support through existing independent agencies, was developed in collaboration with government agencies and advisors. Governance and clinical oversight committees and processes were established. The SPARX program was rapidly adjusted from the CD-ROM version used in the initial RCT for web-based delivery with minor adaptations as outlined in Textbox 1. Promotion was undertaken, including presentations at meetings of health professionals and school staff and via social media advertising targeting young people. Potential users visit the website, which includes access to SPARX and an optional anonymous web-based Patient Health Questionnaire adapted for Adolescents (PHQ-A) [27] assessment, which is presented as a “Mood Quiz,” to help users identify whether SPARX might help them. Those whose score on this optional measure indicates no or minimal symptoms are encouraged to try SPARX to develop skills for any future challenges; those who score in the mild to moderate range are advised that SPARX may be a helpful tool for them; and those who score in the moderately severe or more severe range or who indicate a risk of self-harm are advised to seek further support and try SPARX once they are safe. Those who wish to access SPARX (with or without completing the “mood quiz” first and regardless of “mood quiz” results) must register by entering an email address, creating a username and password, and answering brief demographic questions. They may opt to get automatic reminders by text or email and must agree to the terms of use. Once registered, users may begin the program on a PC or laptop, or, from 2018, on a mobile device. As users begin the first module of SPARX, they complete the first in-program PHQ-A and then progress through SPARX modules in the prescribed order at their own pace. In module 1, users are welcomed to the program, advised to complete 1‐2 modules per week, and provided information on where to get extra help. Automatic email reminders are sent 2 and 4 weeks after the last use of SPARX for those who opted in for reminders and who had not completed the program. The following key changes were made to SPARX from the research version delivered by CD-ROM in the 2012 RCT [7], for the national implementation of the program (Textbox 1).

Textbox 1. **Changes to SPARX (Smart, Positive, Active, Realistic, X-factor thoughts) for national web-based implementation.**

- A Likert scale for rating mood in all modules in the research version was replaced with the Patient Health Questionnaire Adapted for Adolescents (PHQ-A) toward the beginning of modules 1, 4, and 7.
- A list of helping resources and prompts for how and where to seek help was provided on the SPARX website.
- Further messages to prompt help-seeking were added for those with high or deteriorating scores on PHQ-A or item 9 (indicative of a risk of self-harm) within SPARX.
- A dedicated free phone and text number for SPARX users was established. This was supported by a partner telephone and text counseling organization to provide a full-time backup crisis service. Since 2017, users can also request chat-based support via the SPARX website (this is also provided by the partner agency).
- A summary screen for each module was added. This can be printed out and replaces a paper notebook provided with each CD-ROM in the initial SPARX trials.

There were logistical challenges in the first years of implementation, and technical updates were made over this time period. Initially, users were required to download a large file to a computer (not a mobile device); these files were blocked by some firewalls, and downloads were very slow in some settings. Changes in the interface between internet browsers and “Unity Player” (ie, the game platform used for SPARX) meant that only some internet browsers could be used in some time periods. Hence, some registrants could not begin SPARX. A version which could be played on mobile was first available in 2018; however, it appeared as designed for computers and was hard to read and manipulate on mobile devices. A significant update, SPARX (version 2.0), optimized for mobile use, became available after this study period in October 2021. Future analyses will compare SPARX (version 2.0) to data reported here.

### Variables

Study variables were collected from routinely gathered user data and automatically collected analytics. User data requirements had been established with the Ministry of Health in order to allow service evaluation while minimizing barriers to engaging with SPARX. These were self-reported demographics (age, gender, ethnicity, and New Zealand region) and how registrants found out about SPARX (eg, school or health professional), all entered by the user on registration, and the adolescent-modified Patient Health Questionnaire 9-item depression scale (PHQ-A) [27], embedded at the beginning of modules 1, 4, and 7 only. There were no symptom measures in other modules, as youth consultation had indicated potential negative impacts of repeated measures on engagement. The PHQ-A is a robust tool for depression screening [27]. Response options range from never to nearly every day (scores of 0‐3) and although not a diagnostic test per se, the total scores can be grouped into 5 symptom levels: none (0‐4), mild (5-9), moderate (10-14), moderately severe (15-19), and severe (20-27). We report demographic data with gender categorized as female, male, or transgender or intersex or gender not specified; ethnicity categorized using the New Zealand census ethnicity prioritization method; and age categorized into 12‐15 and 16‐19 years. Automatically collected analytics are registrations (registering or signing up for SPARX), opening of the SPARX program (ie, “starting,” or opening the program at least once, for any length of time), start and finish times for each module, and number of modules completed. Our primary outcome variable was PHQ-A score.

### Statistical Analyses

Summaries of continuous variables have been presented as means and standard deviations or medians and interquartiles for skewed data, while categorical variables have been presented as frequencies and percentages. The extent of the skewness was quantified using Pearson’s coefficient of skewness (3×(mean–median)/SD). We interpreted values >0.5 as indicating strong positive skewness. For users who completed at least 2 of the 3 PHQ-A scores, the mean changes in PHQ-A scores from baseline (module 1) to module 4, module 7, and the last available module (4 or 7) were analyzed using paired sample *t* tests. The data were treated as normally distributed due to the number of users included in the analyses [28]. Effect size was calculated by dividing the mean difference by the standard deviation of the difference (Cohen *d* for paired samples *t* test). Data were analyzed with Statistical Package for the Social Sciences (SPSS; version 28.0) and the Statistical Analysis System (SAS; version 9.4).

## Results

Between April 2014 and September 2021, 21,310 adolescents aged 12 to 19 years registered for SPARX. As shown in Table 1, the majority (n=13,593, 63.8%) were female. Registrants could select as many ethnic identities as applied to them, with one ethnicity assigned for the purposes of analysis using the New Zealand census ethnicity prioritization method [29]; 15.7% (n=3354) were Indigenous Māori, 4.6% (n=985) identified as Pacific, 8.4% (n=1799) as Asian, 62.8% (n=13,376) were New Zealand European; and 8.4% (n=1796) were of another ethnicity. Two-thirds (n=14,297, 67.1%) were 12-15 years of age. In total, 13,564 (63.7% of registrants) started SPARX (“starters”). There were significant differences in gender, ethnicity, age, and referral source for starters compared to registrants, with female, Māori, younger, and school-referred participants forming lower proportions of starters than they did of registrants (Table 1). The mean PHQ-A at the start of the program (beginning of module 1) was 13.5 (SD 7.7), with 37.4% (n=4609) reporting mild or moderate symptoms (the target group), 46.7% (n=5742) reporting more severe symptoms, and 16.1% (n=1980) reporting no to minimal symptoms, as shown in Table 1. School was the largest single point of referral to SPARX, with 32.0% (n=4340) of those who started and a professional (ie, counselor, doctor, or nurse) referral was second, with 29.8% (n=4047).

**Table 1.** Characteristics of 12‐ to 19-year-olds registering for and starting SPARX (Smart, Positive, Active, Realistic, X-factor thoughts).

	Total	Started SPARX		
	Registrations	Yes	No	Difference *P* value
Total, n	21,310	13,564	7746	—^a^
Gender, n (%)				<.001
Female	13,593 (63.8)	8499 (62.7)	5094 (65.8)	
Male	6507 (30.5)	4265 (31.4)	2242 (28.9)	
Transgender or intersex or gender not specified	1210 (5.7)	800 (5.9)	410 (5.3)	
Prioritized ethnicity, n (%)				<.001
Māori	3354 (15.7)	1941 (14.3)	1413 (18.2)	
Pacific	985 (4.6)	538 (4.0)	447 (5.8)	
Asian	1799 (8.4)	1202 (8.9)	597 (7.7)	
Other	1796 (8.4)	1142 (8.4)	654 (8.4)	
New Zealand European	13,376 (62.8)	8741 (64.4)	4635 (59.8)	
Age				<.001
12‐15 years, n (%)	14,297 (67.1)	8908 (65.7)	5389 (69.6)	
16‐19 years, n (%)	7013 (32.9)	4656 (34.3)	2357 (30.4)	
Mean (SD)	14.8 (1.9)	14.9 (1.9)	14.6 (1.9)	<.001
Referral source, n (%)				<.001
School	7136 (33.5)	4340 (32.0)	2796 (36.1)	
Counselor, doctor, or nurse	5924 (27.8)	4047 (29.8)	1877 (24.2)	
Friend	1370 (6.4)	887 (6.5)	483 (6.2)	
Google, Facebook, or advertisement	3090 (14.5)	1842 (13.6)	1248 (16.1)	
Other	3790 (17.8)	2448 (18)	1342 (17.3)	
Baseline PHQ-A^b^, n (%)				—
Did not complete	—	1248 (9.2)	—	
Not depressed (0‐4)	—	1980 (16.1)	—	
Mild (5-9)	—	2061 (16.7)	—	
Moderate (10-14)	—	2548 (20.7)	—	
Moderately severe (15-19)	—	2523 (20.5)	—	
Severe (20-27)	—	3219 (26.1)	—	

a Not applicable.

b PHQ-A: Patient Health Questionnaire adapted for Adolescents.

**Figure 1.**
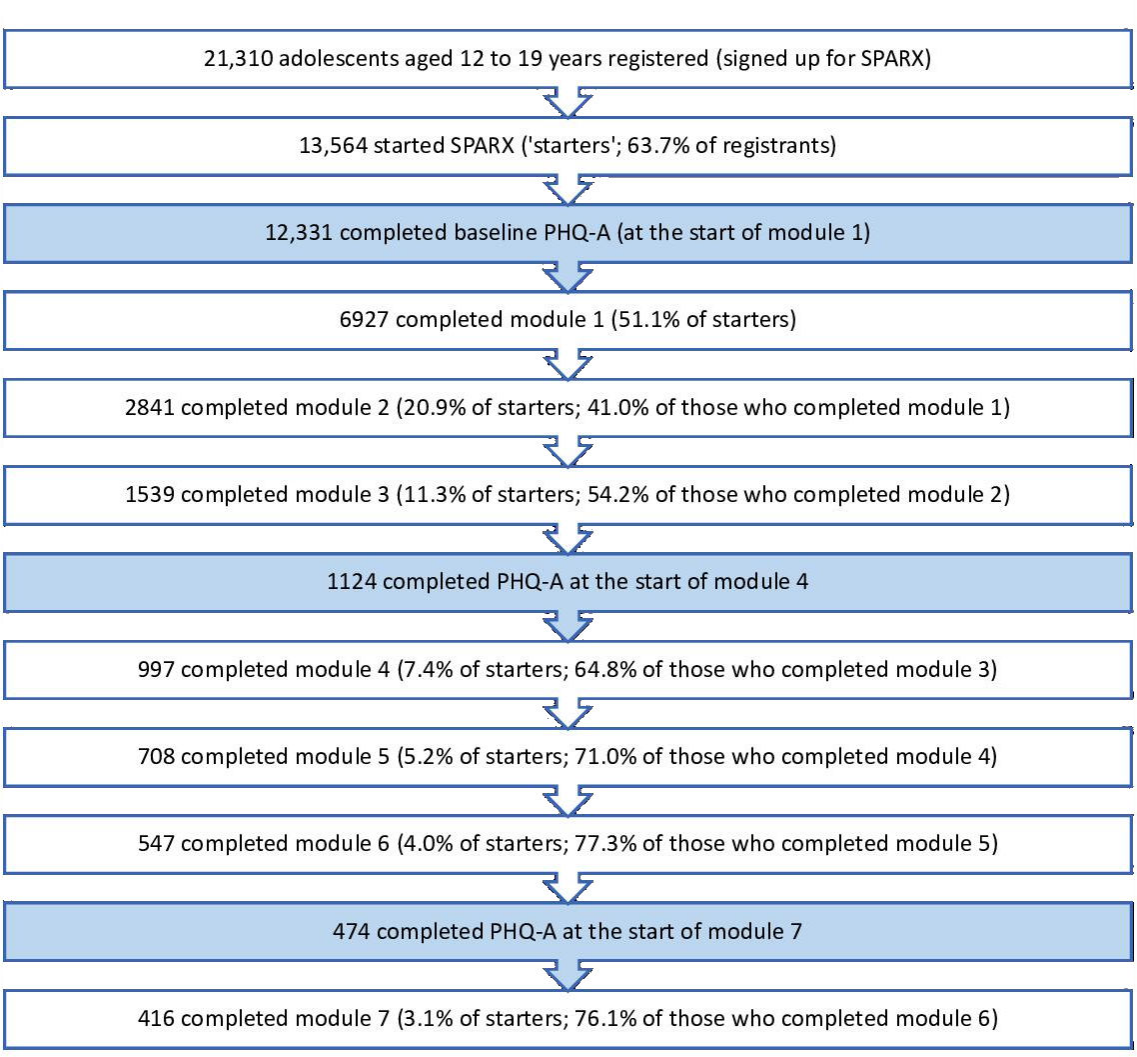
Engagement, April 2014 to September 2021. SPARX: Smart, Positive, Active, Realistic, X-factor thoughts; PHQ-A: Patient Health Questionnaire adapted for Adolescents.

Of those who began SPARX (“starters”; n=13,564), 51.1% (n=6927) completed module 1; 20.9% (n=2841) and 11.3% (n=1539) completed modules 2 and 3, respectively; 7.4% (n=997) completed module 4 and 3.1% (n=416) finished all 7 modules, as shown in Figure 1. These data represent a 41.0%‐77.3% retention at each module (also shown in Figure 1). Users spent a median time of 19 minutes per module, with an interquartile range of 15-25 minutes. Among those who started the last module and have the module start time available (n=435), the median completion time was 12 days, with a wide interquartile range of 2-42 days (Figure 1).

Table 2 provides the mean PHQ-A scores for modules 1, 4, and 7 and paired difference means. Paired *t* tests show significant reductions in PHQ-A scores between module 1 and module 4 and between module 1 and module 7, as well as module 1 to the last available measure (module 4 or 7), with *P*<.001 for each of these. Effect sizes (Cohen *d*) were 0.38 for those who completed the second PHQ-A only (at module 4); 0.51 for those who completed the PHQ-A at module 7; and 0.43 for baseline to last available score (module 4 or 7).

**Table 2.** PHQ-A^a^ score at baseline and module 4 or 7, including paired samples *t* tests.

	Participants, n	Module 1 (baseline), mean (SD)	Comparison level, mean (SD)	Paired difference, mean (SD)	*P* value	Effect size
Modules 1-4	1124	14.0 (7.4)	11.8 (7.9)	2.2 (5.7)	<.001	0.38
Modules 1-7	473	14.1 (7.3)	10.5 (8.5)	3.6 (7.0)	<.001	0.51
Module 1 to last available (module 4 or 7)	1124	14.0 (7.4)	11.2 (8.1)	2.8 (6.5)	<.001	0.43

a PHQ-A: Patient Health Questionnaire adapted for Adolescents.

## Discussion

### Principal Findings

This article is among the first to analyze national implementation data from an evidence-based self-help intervention for adolescent depression. We present data on reach, engagement, and impact among the target age group (12- to 19-year-olds) from the first 7 years of availability, beginning when SPARX was first offered without referral and at no cost to anyone with a New Zealand IP address. Between 2014 and 2021, approximately 2% of the national adolescent population [30] registered for SPARX, with strong uptake by Indigenous Māori. Participants who completed 2 or more PHQ-A assessments showed moderate improvements in depressive symptoms, with effect sizes comparable to face-to-face therapy [31-33] and consistent with findings from the original RCT of SPARX [7]. Despite the RCT being a pragmatic trial with minimal exclusion criteria and limited additional user contact beyond usual practice [7], real-world use of SPARX differed in key ways; users varied in baseline symptom severity, progressed through the program at different paces, and completed the program at significantly lower rates. These differences highlight the complexities of implementation and the need to address real-world barriers to engagement. Two decades of research have shown that evidence-based digital therapies can be both effective and engaging in controlled settings [34,35]. However, implementation in real-world contexts remains a critical challenge. Analysis of implementation data offers important insights for informing improved impact in communities [35-39].

We have demonstrated that it is feasible to shift from the research setting to real-world implementation in a relatively short time period (ie, going from the publication of the original RCT findings in late 2012 to national roll-out in 2014) and to achieve sizeable population reach. The registration numbers demonstrate interest in the program. While many of those who registered did not begin the intervention, this still represents a major increase in access to evidence-based therapy at a national level. Many interventions fail to reach Indigenous and minoritized youth. The data presented here demonstrate interest from Māori young people. Although given the disproportionate burden of distress experienced by Māori [40], ongoing recruitment efforts are indicated for improving equity. Further efforts are also indicated to reach Pacific adolescents, given the uptake reported was low compared to population numbers and reported distress among Pacific youth [41]. Gender minority adolescents, including transgender and gender-questioning youth, form between 1% and 4% of the New Zealand secondary school population [42], with many reporting depressive symptoms and poor access to care [42]. It is pleasing to see this group having comparatively high engagement with SPARX (eg, 207 transgender adolescent registrations) [43].

The retention of effect size from the RCT to unsupported implementation, as observed in individuals who undergo at least two assessments, is a notable finding. The efficacy of interventions often diminishes when applied in routine practice [44,45]. The use of computerized programs, which automate delivery, can mitigate decreases in treatment fidelity. Extensive meta-analyses and systematic reviews have established that computerized or internet-delivered therapies for depression can be as effective as face-to-face treatments under research conditions [6,46]. The effect sizes reported in this study underscore treatment effectiveness in the real world. However, there are important caveats. First, these are correlational findings that apply to those completing 2 or more assessments. Second, despite effect sizes matching or exceeding the average for psychotherapeutic treatments for adolescent depression [47], mean postintervention scores remained clinically significant (over the cutoff on the depression measure). These findings emphasize the importance of integrating evidence from both controlled studies and real-world implementation research. They also highlight the ongoing need to enhance the effectiveness of treatments for depression [47].

There were important differences in how public registrants used SPARX compared to the advice given on the website and within the program. Less than half of 12‐ to 19-year-old users (n=4609, 37.4%) were in the target group of mild to moderate symptoms of depression. Almost half reported more severe symptoms (n=5742, 46.7%) and hence were automatically advised to get more support and then use SPARX alongside that extra help if desired. We do not know if they did this. Usage also diverged widely from the recommended rate of 1 to 2 modules per week, with most users working through the program much more quickly. It is important to note that users may not use internet-based programs as intended by developers, and hence to adjust communications to users or to adjust the programs themselves to fit user behavior if needed. Adaptive, user-centered approaches (ie, where programs are adjusted to fit user behavior and requirements) are common in the development of digital tools and ensure that interventions evolve to meet user behavior rather than rigidly enforcing compliance with established protocols.

Adherence in this study was considerably lower than in the 2012 RCT, where 86% of those randomized to SPARX completed at least module 4 and 60% completed all 7 modules [7]. Reduced adherence is not unusual in the translation of interventions from research to real-world settings [48], as is seen in digital tools for mental health [18,49] and also in other areas, including face-to-face therapy and medication [26,50,51]. For example, 0.5% of MoodGYM users completed a final assessment in the program as implemented, compared with 22.5% of participants in a trial of the same program [52]. Similarly, routinely collected internet traffic data shows that median retention in publicly available mental health apps is less than a quarter of that reported in trials [18]. Baumel et al [53] propose that retention in digital self-help apps and programs should be considered in the context of their low barriers to entry. In contrast to the complexities of accessing face-to-face therapies, which may require considerable effort to access, web-based self-help tools can often be accessed without a referral or an appointment, at any time, often within minutes and at no or low cost. Users may explore multiple tools before selecting one, and some may be “just looking” without the level of concern or motivation involved in getting to a clinical service [53].

Limited retention may be a shortcoming of digital tools, but alternatively, brief engagement might be all that some users need at the time. For example, those who complete only the first module of SPARX still receive key psychoeducation and may benefit from understanding that they are not alone, that there are ways to improve mental health, and that further help is available through the program or other sources. Relatedly, there is evidence of benefits of very brief psychotherapeutic input, where single-session therapies can be helpful [54]. Nevertheless, engagement rates reported here are disappointing. SPARX has not been designed or evaluated as a single-session intervention.

Some limitations in engagement are likely to reflect logistical challenges. For example, users who only had access to a mobile device or who had slow internet speeds would not have been able to begin SPARX in the first years. Second, while the therapeutic content of SPARX had been carefully evaluated, there were opportunities for an improved computer interface and human-computer interaction, for example, in making movement of characters within the game easier and having increased save points within the game. Further, the presentation of SPARX as “a game” may have increased appeal for some adolescents by providing a non-threatening and engaging approach [55]; however, users accustomed to high-budget, rapidly evolving commercial games may have been disappointed [56,57]. These challenges highlight the importance of ongoing technological updates, regular user experience reviews, and ongoing attention to communications about digital tools to target users. An updated version, SPARX 2.0, with enhanced graphics and gameplay and an updated website, was launched in 2023, and data will be reported in future analyses. Further analyses of SPARX onboarding and user experience are underway. Blended care or guided approaches, where iCBT is provided alongside face-to-face therapy or with a therapist or e-coach input by email, text, or telephone support, typically (although not always) yields improved retention in comparison to pure self-help [58]. However, these options also bring increased costs and may reduce appeal for those who want anonymous help or do not want to talk to professionals, an important consideration given so many adolescents with depressive symptoms do not seek professional help [5].

There are important limitations in this study. In total, 7 years of data have been presented. However, this is an observational study: the data are within subject; as such, there is no comparison group. Importantly, the results are correlational; findings may be confounded by spontaneous improvements in PHQ-A scores. Further, it is possible that the participants who were particularly motivated were more likely to both improve and to complete a higher number of modules of SPARX. These findings should, therefore, be considered alongside those from experimental studies with control groups. There is also no follow-up of those who did not engage, and there is only self-reported data pertaining to a single screening measure. Adolescents who completed the PHQ-A on the SPARX website and then immediately began SPARX would have completed the measure twice within a short time, potentially affecting their responses. While the authors include the co-developers of SPARX or those directly involved in its dissemination, the study plans and results have been reviewed by advisory groups. Implementation issues arose that were specific to this time period and this particular intervention; hence, caution should be exercised in considering implications for other settings. At the same time, there are few studies of this nature, and the findings highlight valuable opportunities for improving the real-world impact of digital interventions.

We have shown that it is possible to roll out iCBT interventions nationally for adolescent depression and significantly increase access to evidence-based therapies in the population. However, this requires ongoing efforts to optimize impact. Reach can be supported by ongoing promotion to users and stakeholders via advertising, training, relationships with providers, and local champions [38]. Improving engagement also requires continued attention. There are many opportunities here, including the ethical use of artificial intelligence. There are examples of effective or promising approaches in the use of single-session interventions [54,59]; the use of brief, just-in-time “micro interventions” [53]; increased use of social or automated support including chatbots, chatrooms, artificial intelligence, and text support [38]; enhanced personalization [49] and cultural responsiveness [38]; developments in telepresence and gamification [60]; increased use of persuasive design [61]; strong co-design, including increased building of digital tools to match how young people use the internet for their own well-being [62]; increased use of promotions, marketing, and champions [38]; and increased integration as part of pathways to care and mental health ecosystems [63]. Rigorously testing these innovations in research settings *and* analyzing and reporting implementation data will be important for rapid and ethical development in the field and to assist in improving mental health outcomes in communities.

### Conclusions

Given the barriers to mental health treatment for young people, free and private access to evidence-based digital therapy is essential. Programs that can be made freely available to large numbers of users around the clock are critical components of a comprehensive contemporary mental health system. To optimize the impact of these tools, implementation data should be routinely reported, and ongoing promotion, updates, and development should be prioritized. We propose that providers of evidence-based digital tools, such as iCBT, should routinely report implementation data, and not trial data alone, so that the sector can understand and optimize the health impacts of digital mental health interventions in the real world.

## Abbreviations

CBT: cognitive behavioral therapy
iCBT: internet-based cognitive behavioral therapy
IP: Internet Protocol
PHQ-A: Patient Health Questionnaire adapted for Adolescents
RCT: randomized controlled trial
SPARX: Smart, Positive, Active, Realistic, X-factor thoughts
STROBE: Strengthening the Reporting of Observational Studies in Epidemiology
